# Metronidazole for the treatment of cutaneous vulval Crohn disease: A systematic review

**DOI:** 10.1002/ski2.210

**Published:** 2023-04-26

**Authors:** Nina Simon, Zahra Moledina, Rosalind Simpson, Lisa Kirby

**Affiliations:** ^1^ University of Nottingham School of Medicine Nottingham UK; ^2^ Department of Dermatology Nottingham University Hospitals NHS Trust Nottingham UK; ^3^ Centre of Evidence Based Dermatology, School of Medicine University of Nottingham Nottingham UK

## Abstract

**Background:**

Cutaneous vulval Crohn disease (VCD) is an under‐recognised extra‐intestinal manifestation of Crohn disease (CD) which is challenging to identify and treat. It causes significant oedema, painful deep fissures, and has potential to cause permanent disfiguring changes to vulval anatomy. There is no agreement on the best management for VCD.

**Objectives:**

This systematic review evaluates the use of metronidazole for the treatment of VCD in women and children.

**Methods:**

We conducted a systematic review (PROSPERO CRD42021285033) of the use of metronidazole in clinically or histologically diagnosed non‐contiguous VCD in patients of all ages and ethnicities. We recorded clinical improvement, reduction in flares, relapse and adverse events using a standardised form.

**Results:**

49 records (40 case reports and 9 case series) met inclusion criteria, comprising a total of 57 patients with an age range of 5–61 years. The most reported presenting features in VCD were: oedema, erythema, ulcers/fissures and induration/thickening. Gastrointestinal CD was present in 33/57 (58%). Vulval biopsies were undertaken in 47/57 (83%). Daily doses ranged from 250 to 1500 mg with treatment duration 8 days to 18 months. Improvement of any magnitude was observed in 40/57 (70%) cases. Relapse was described in 11/57 (19%) cases. No response/worsening was reported in 17/57 (30%) cases. Adverse events occurred in two patients.

**Conclusion:**

Metronidazole appears to be useful in managing VCD, either as a primary treatment or adjunctive therapy. However, the evidence is insufficient for firm conclusions to be drawn. Further studies including randomised controlled trials are recommended.

## Graphical abstract

Vulval Crohn disease is a devastating chronic condition causing irreversible skin changes. It is underrecognised and a challenge to treat. We have undertaken a systematic review of the use of metronidazole for the treatment of cutaneous vulval Crohn disease. We summarise the presenting features and histological findings, to provide a broad overview of the condition.

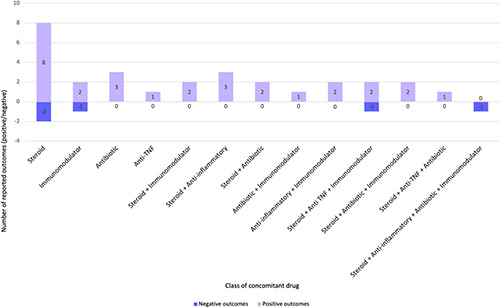

1

**What is already known about this topic?**

- Vulval Crohn Disease (VCD) is a cutaneous manifestation of Crohn disease (CD) and can affect paediatric and adult females
- Diagnosis and treatment of VCD is difficult and may result in the under‐reporting of the condition

**What does this study add?**

- This systematic review evaluates the evidence for metronidazole as a treatment for VCD, whilst also reviewing patient demographics, diagnostic features and association with gastrointestinal CD.
- Metronidazole may be useful as a primary or adjunctive treatment for VCD, but the existing evidence is of low quality and firm conclusions cannot be drawn.
- Diagnosis of VCD may be aided by being vigilant for clinical features and the knowledge it can either occur on its own or with gastrointestinal CD. Non caseating granulomas are a hallmark histological feature, but these may be absent.

## 2 INTRODUCTION

Crohn disease (previously termed ‘Crohn's disease’)^1^ is a chronic inflammatory granulomatous disorder commonly affecting the gastrointestinal tract. Extra‐intestinal cutaneous manifestations occur in approximately 10% of all patients with Crohn disease (CD)^2^ and include erythema nodosum, pyoderma gangrenosum, fissures and abscesses.^3^

Since first described by Parks et al. in 1965, less than 200 cases of cutaneous vulval Crohn disease (VCD) non‐contiguous with the gastrointestinal tract, also known as ‘metastatic’ CD have been reported.^4^, ^5^ This contrasts with the more common complex fistulating CD contiguous with the gastrointestinal tract.^2^

The pathogenesis of metastatic CD is not fully understood and the origin of skin lesions in VCD remains unknown. Several causes have been suggested, which include, but are not limited to, the deposition of immune complexes from the gastrointestinal tract^6^ and type IV hypersensitivity reactions with cross reaction to antigens of the skin.^7^ However, no definitive cause has been universally accepted.

**FIGURE 1:**
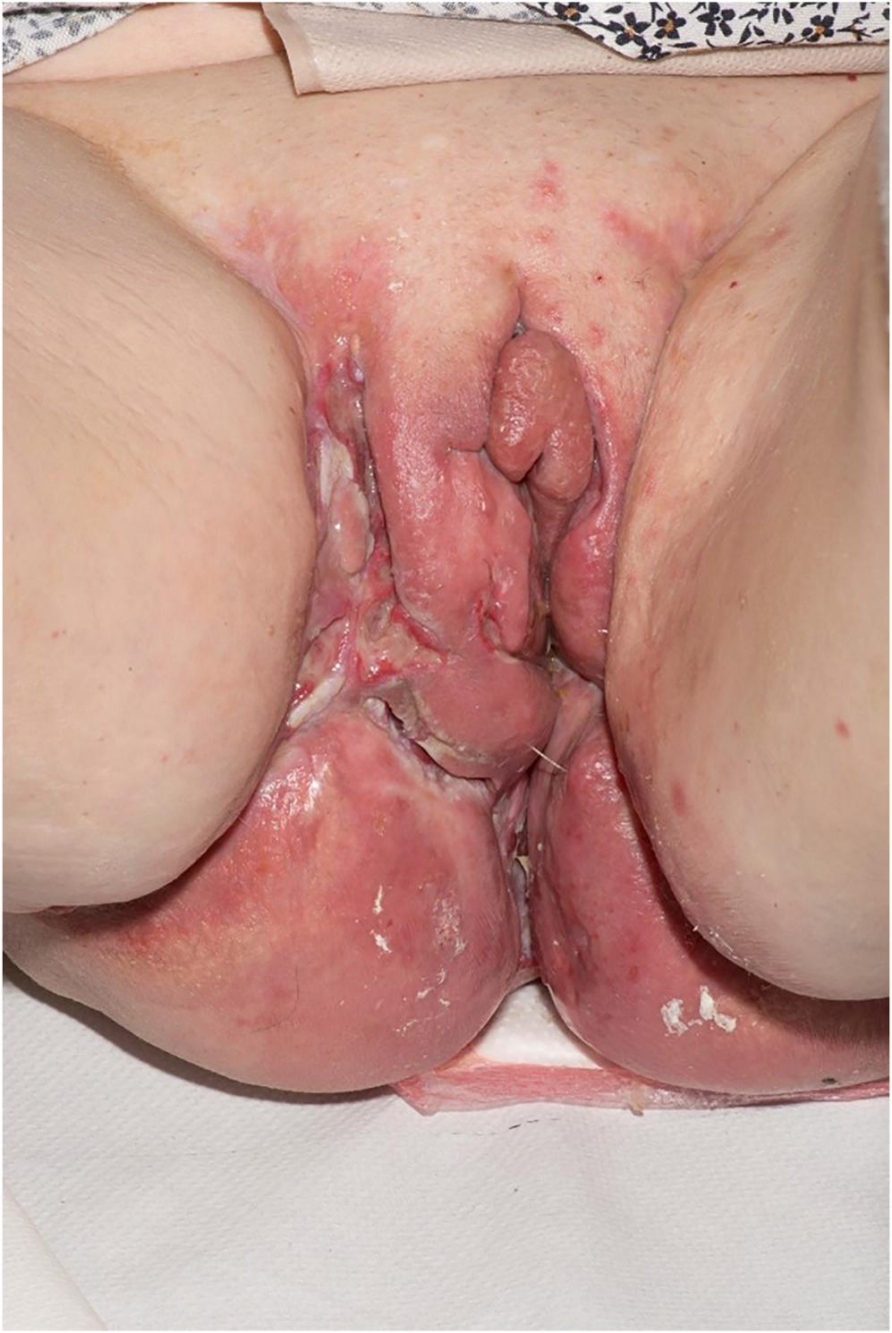
Presenting features include knife cut ulcers and fissures, oedema and dusky violaceous discolouration.

**FIGURE 2:**
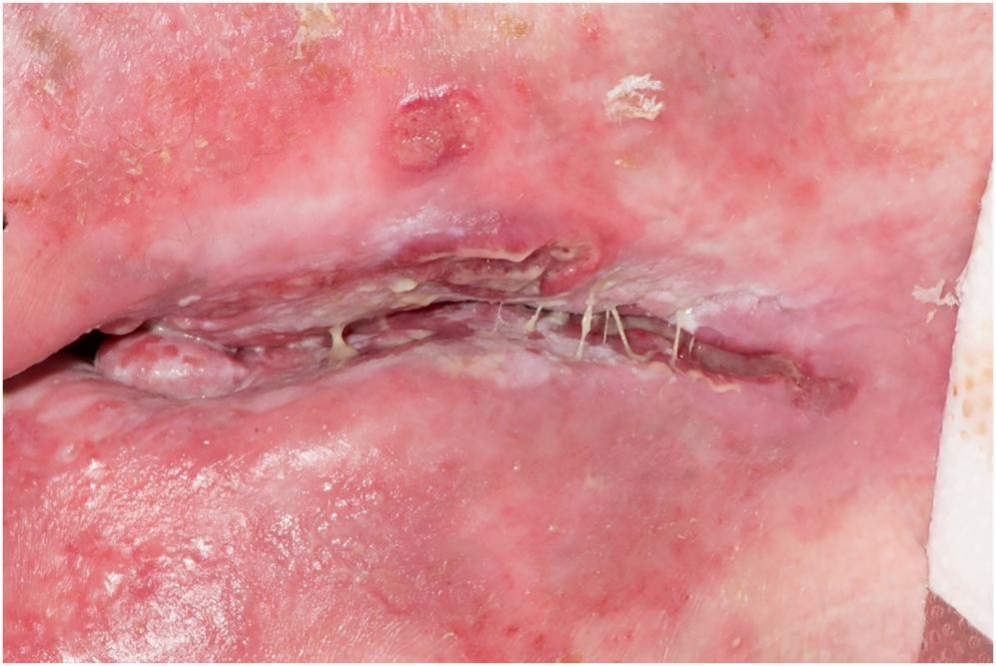
Knife cut ulcers and fissures.

Common presenting features of VCD include ‘knife cut’ ulcers, lesions, swelling or oedema which can affect any part of the vulva from the labia minora or majora to the vaginal wall (see Figures 1 and 2). Typically, the ulcers are painful, although they can occasionally be asymptomatic.^5^ Due to similarities with other conditions, metastatic CD is hard to diagnose. Hidradenitis suppurativa (HS) is the main differential and the two conditions can co‐exist. Diagnosis of VCD is usually made on correlation of clinicopathological findings. Non‐caseating, giant cell granulomas on histology are strongly supportive but the over‐reliance on their presence may lead to underdiagnosis.^8^

VCD is difficult to treat. Therapeutic options include corticosteroids, antibiotics, aminosalicylates, immunomodulators, biologics and surgical management. Due to the relative lack of literature regarding VCD, no treatment guidelines have been published and treatments for the condition are mostly extrapolations from those used for gastrointestinal CD.

Metronidazole has been reported as having therapeutic benefit. If demonstrated to be effective, it is a potentially good treatment option for VCD, due to its relative low cost and overall safety profile. A side effect (frequency not known) to be aware of is possible development of peripheral neuropathy with long term or intensive therapy.^5^, ^9^, ^10^

The aim of this systematic review was to evaluate the evidence for metronidazole as a treatment for patients with a clinical or histological diagnosis of VCD.

## 3 METHODS

This review was registered with the Prospective International Register for Systematic Reviews (PROSPERO) number CRD42021285033 and reported in line with PRISMA guidelines (Appendix 1). Pubmed, MEDLINE and Embase were searched in August 2022 from inception. Keywords were ‘vulva’ or ‘vulv*’ or ‘genitalia’ or ‘genit*’ or ‘perineum’ or ‘perianal’ and ‘Crohn disease’ or ‘Crohn*’ and ‘metronidazole’ or ‘metro*’. A full search strategy is available (Appendix 2). Grey literature sources and articles in languages other than English were excluded.

### 3.1 Eligibility

All study types were included. Included studies were required to have a clear definition of the study population (adult and paediatric females with VCD as diagnosed clinically or histologically), and documented use of metronidazole treatment and outcome. Exclusion criteria were studies involving males (where data for females could not be extracted separately) and those focussing on fistulating CD. Studies that did not include original research data and articles in languages other than English were excluded.

### 3.2 Data extraction

A standardised extraction form was used to extract data. Two review authors (NS, ZM) extracted data independently and any discrepancies were identified and resolved through discussion with two further authors (LK, RS). Data on year of publication, country, study type, declared conflicts of interest and number of participants were extracted. Variables recorded were: patient age, presenting symptoms, method of diagnosis (clinical or histological), gastrointestinal involvement, disease duration, concomitant treatments, duration of follow‐up, metronidazole regimen, patient response to metronidazole, relapse and adverse events.

### 3.3 Quality assessment

Methodological quality (risk of bias) was assessed and recorded independently by two review authors. The Joanna Briggs Institute checklist was used.^11^ Case series and case reports are automatically considered to be low quality evidence. If studies meet <50% of the checklist criteria, they are graded as ‘very low’ quality.

## 4 RESULTS

**FIGURE 3:**
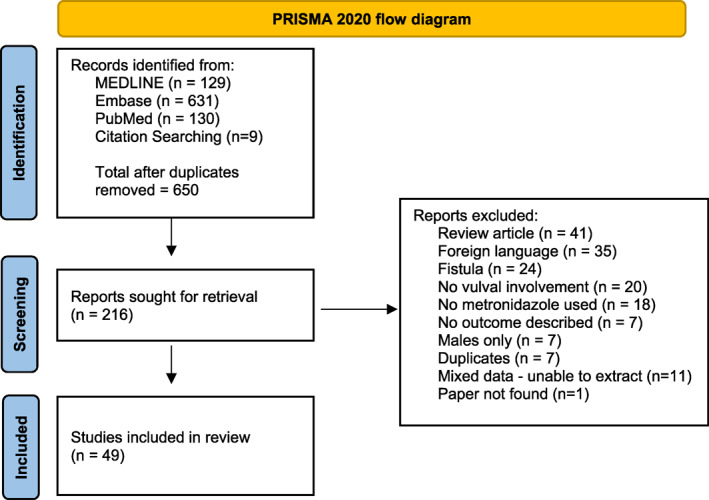
PRISMA Flow diagram.

The initial search identified 650 records of which 49 met inclusion criteria (Figure 3). These comprised 40 case reports and 9 case series consisting of 57 patients with VCD treated with metronidazole. Appendix 3 summarises the identified study characteristics, risk of bias and results of individual studies. Due to the nature of the studies identified and because no randomised controlled trials were found, meta‐analysis was not possible and the results are reported descriptively.

### 4.1 Age and clinical presentation

The most commonly reported presenting features in the cases included in this review were swelling/oedema (*n* = 35) and redness/erythema (*n* = 24). Further presentations included ulcers/fissures (*n* = 17), induration/thickening (*n* = 14), pruritis (*n* = 10), dusky/violaceous discolouration (*n* = 4), vaginal discharge (*n* = 4) and papules/nodules (*n* = 3). The age of affected patients from identified articles ranged from 5 to 61 years, with mean and median ages 25 and 23 years respectively. Nineteen patients were aged 16 years or younger.

### 4.2 Histopathological diagnosis

Vulval biopsies were undertaken in 47 out of 57 cases although this number may be higher since there was no documentation of vulval biopsy in 8 cases. Of the remaining two cases, one patient declined vulval biopsy, and in the other, VCD diagnosis was presumed following histopathological diagnosis of gastrointestinal CD. There were two cases of patients diagnosed with VCD in the absence of non‐caseating granulomas. In both, a perivascular inflammatory infiltrate was detected which was further described in one case as consisting of lymphocytes, epithelioid histiocytes and occasional plasma cells.

### 4.3 Metronidazole administration and concomitant treatments

**FIGURE 4:**
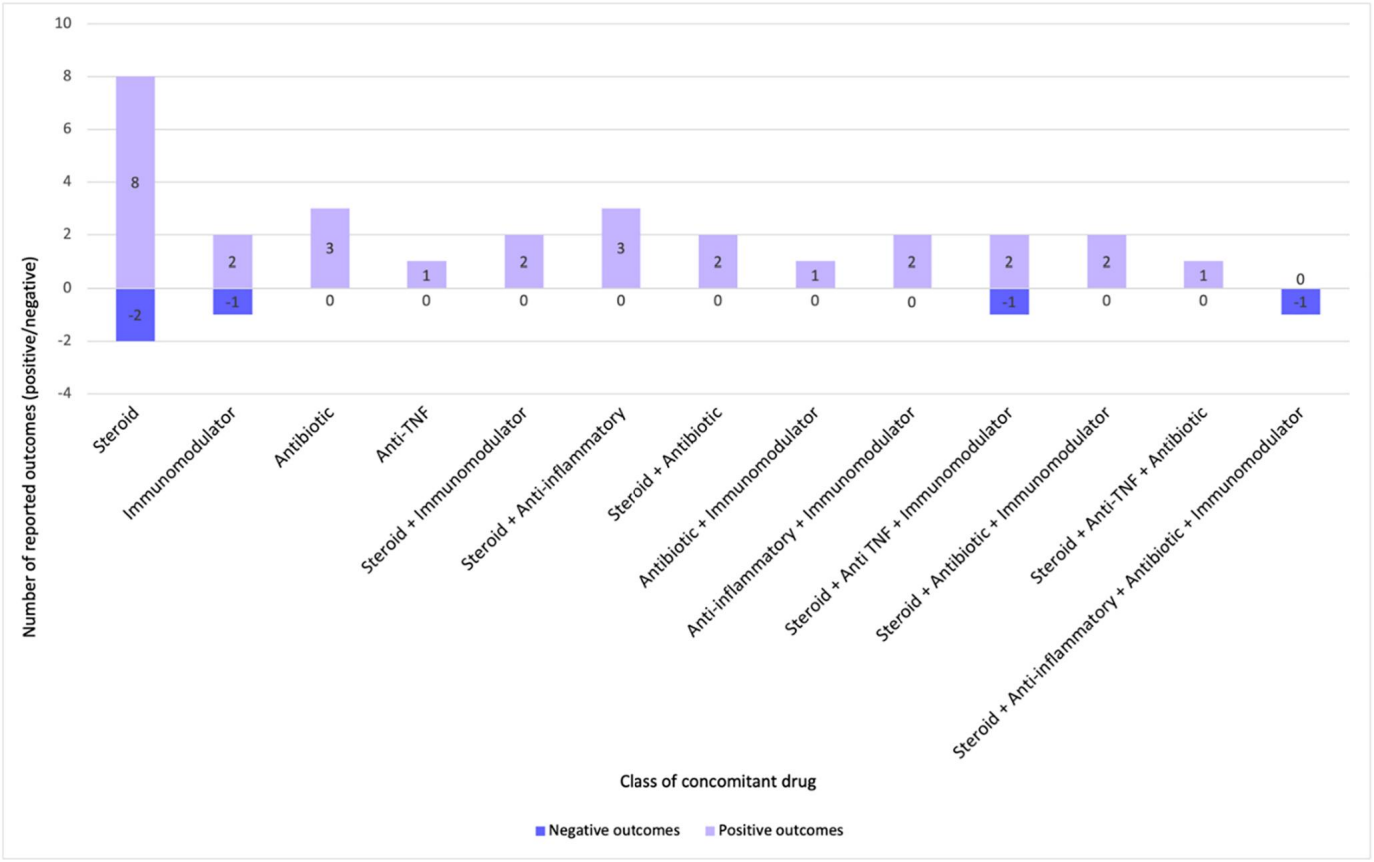
Frequency and outcomes of treatments given in combination with metronidazole. Positive outcomes were defined as clinical or symptomatic improvement of any magnitude, and negative outcomes were no improvement/worsening or inadequate response.

In the 34 cases where administration information was given, metronidazole was administered topically (*n* = 4) and orally (*n* = 30). Daily doses ranged from 250 to 1500 mg. Treatment duration was only documented in 23 cases and ranged from 8 days to 18 months. Treatment with metronidazole was frequently combined with concomitant treatments including oral and topical steroids (*n* = 24), immunomodulators (*n* = 14), anti‐TNF treatments (*n* = 9) and anti‐inflammatory medications (*n* = 6). Further details of these treatments are given in Figure 4.

### 4.4 Response to metronidazole

Positive responses (reported clinical or symptomatic improvement of any magnitude) were documented in 40/57 (70%) cases. Of these, 13 cases described ‘complete’ healing. Thirty of the ‘positive responders’ also received one or more concomitant treatments, most frequently corticosteroids (*n* = 24) and immunomodulators (*n* = 14). Poorer outcomes (no improvement reported, or worsening/inadequate response) were documented in 17 (30%) cases. Four of these patients received one or more concomitant treatments.

### 4.5 Adverse events

There was limited reporting of adverse events in the included studies, with only two cases explicitly described. It is unknown whether side effects to metronidazole occurred in the remaining studies. In one case, a patient treated with 400 mg metronidazole three times daily for a year experienced associated peripheral neuropathy. Treatment was discontinued, however, complete healing of VCD was reported. The patient suffered from a relapse of vulval symptoms and was subsequently treated with azathioprine with minimal improvement. In the second case, the patient had elevated liver enzymes noted after a month of treatment. Response to treatment with metronidazole and need for subsequent investigation and treatment was not documented.

### 4.6 Duration of follow‐up and relapse

As the duration of follow up across studies was often unreported, disease duration and relapse rate cannot be determined with any great degree of confidence. The reported disease duration varied greatly and ranged from 2 weeks to 10 years. Relapse was described in 11 (28%) cases, with five following the reduction or stopping medication. Relapse times ranged from 2 weeks to 1 year. In one case where relapse occurred on weaning metronidazole subsequent lesions no longer responded to metronidazole alone.

### 4.7 Methodological quality

The studies were appraised for quality using the Joanna Briggs Institute checklist for case series and case reports. Twenty two (45%) of the studies were graded ‘Low’ and 27 (55%) studies were graded ‘Very low’.

## 5 DISCUSSION

This systematic review has evaluated the existing evidence for treatment of VCD with metronidazole, whilst also considering diagnosis and clinical presentation of the disease. Vulval disease can have a devastating impact on patients. In a survey carried out by the British Association of Dermatologists (BAD) involving 325 women who had a current or past diagnosis of a vulval health disorder, around 90% of participants stated that their emotional health and mental well‐being had been negatively impacted by their condition^12^ (Figure 4).

**FIGURE 5:**
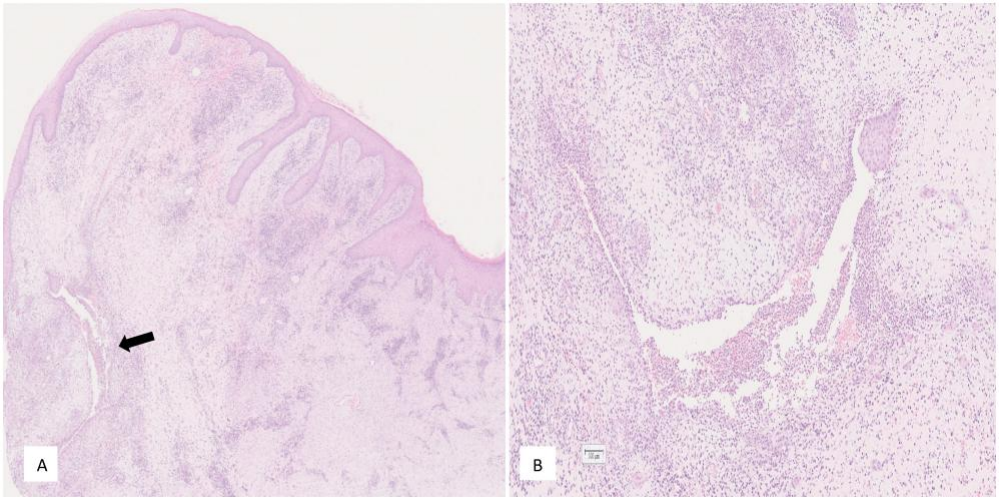
Haematoxylin and Eosin stained sections of vulval skin: (a) Histology shows mild epidermal hyperplasia. The dermis is fibrotic and infiltrated with a mix of acute and chronic inflammation with no granulomas. (b) Sinus tract with denuded epithelial lining and filled with polymorph nuclear leucocytes. Magnifications ×5 (a) and ×20 (b).

The overreliance on histopathological information limited to non‐caseating granulomas may lead to underreporting of VCD. *Foo et al.* observed that reported cases of VCD are generally made with histopathology limited to non‐caseating granulomas.^8^ However, non‐caseating granulomas are absent in approximately half of patients diagnosed with gastrointestinal CD (Figure 5). If this is also the case with VCD, the reliance on histological information may contribute to underreporting and delay in diagnosis. Therefore, granulomas should not be a requisite for a diagnosis of VCD.

**FIGURE 6:**
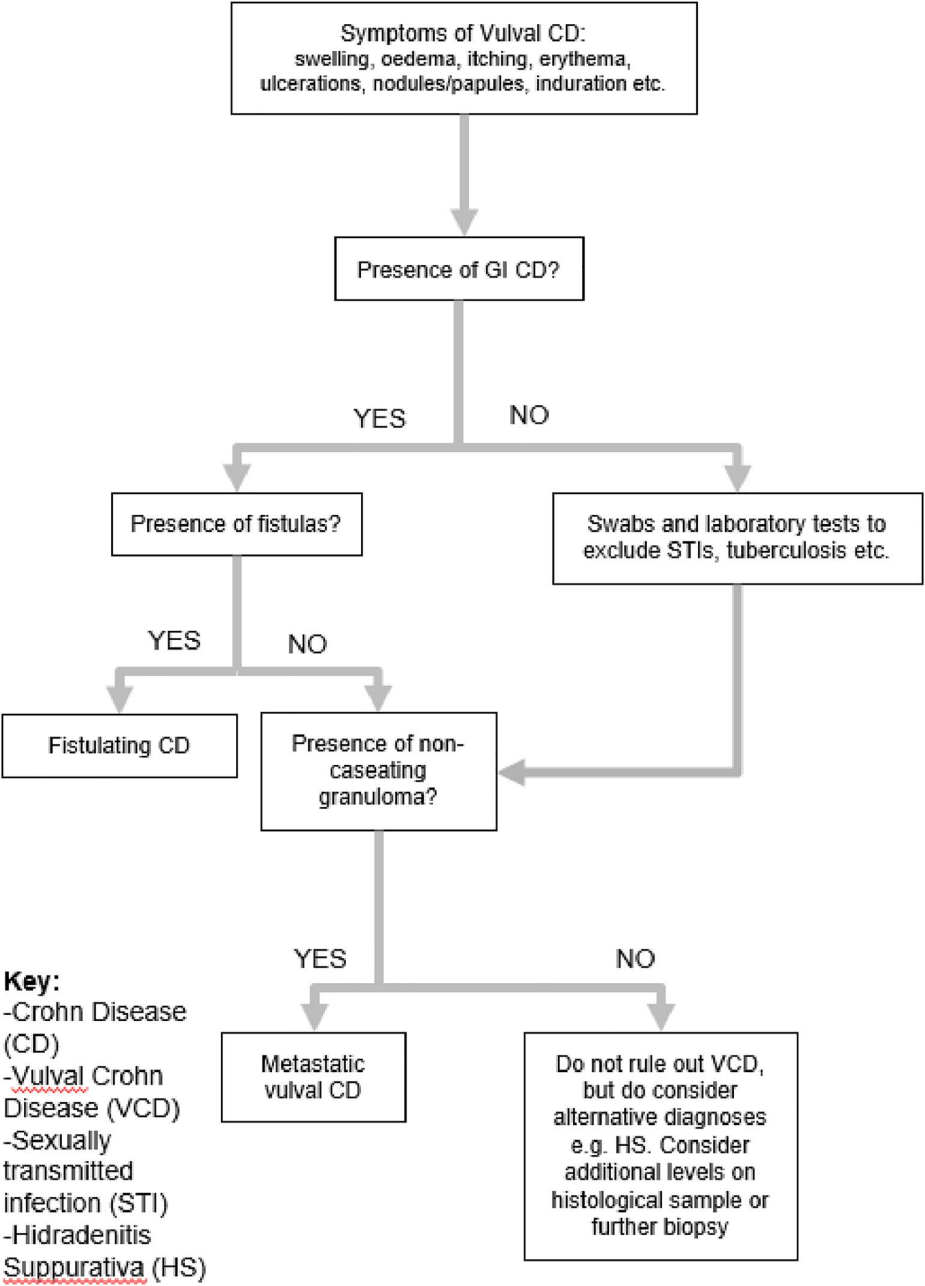
Diagnostic flow diagram.

Since VCD regularly manifests alongside gastrointestinal CD, with 57% of patients diagnosed with VCD in our study also suffering from gastrointestinal CD, we suggest that a starting point in improving detection is exercising vigilance for VCD in patients with gastrointestinal CD. In the alternate, gastrointestinal CD was only diagnosed in five patients following detection of VCD. As such, where a patient is diagnosed with one condition, it is important to consider the other. The authors propose a diagnostic flow diagram (see Figure 6) with factors to consider in diagnosing VCD.

Hidradenitis suppurativa is the main differential diagnosis and clinically may be indistinguishable from VCD. The presence of pseudocomedones and distribution of lesions in other typical sites such as the axillae, groin, perineal and perianal region, buttocks and submammary areas should raise clinical suspicion for HS.

The primary aim of treatment in any chronic inflammatory disorder, including VCD, is to induce and maintain remission. The optimal metronidazole dosing regimen for inducing remission cannot be drawn from this review, although successful alleviation of symptoms was shown for doses as low as 250 mg daily. Most patients were started on metranidazole as an adjunctive treatment for their CD alongside other systemics, hence it is difficult to draw firm conclusions on the efficacy of metronidazole alone.

The mechanism of action of metronidazole in VCD is unclear but is believed to be related to its anti‐inflammatory and anti‐infectious properties.^13^ However, its potential side effects, which include epileptiform seizures and peripheral neuropathy with long term or intensive therapy cannot be ignored^10^ and limit its long‐term use. The British National Formulary recommends clinical and laboratory monitoring if systemic treatment with metronidazole exceeds 10 days.^10^ Adverse effects have only been reported in two studies included in this systematic review. A lack of reporting does not mean adverse events did not occur; rigorous reporting of adverse events is an important consideration for future dermatology studies.

Peripheral neuropathy resulting from metronidazole administration has been documented in patients suffering from perineal CD with one study of 27 patients reporting half of patients experiencing paraesthesia due to peripheral neuropathy following metronidazole treatment.^14^ This subsided upon dosage reduction or discontinuation of metronidazole, sometimes months after the treatment was discontinued. No other major side‐effects were reported. Lack of reporting of side effects in our included studies does not exclude the possibility that these occurred; rigorous adverse event reporting is imperative for future dermatology studies.

Various theories have been suggested for the mechanism by which metronidazole induces peripheral neuropathy although there is no consensus. One theory is that the free radicals produced as a product of metronidazole metabolism are damaging to nerves.^15^ Another theory proffers that nerve axons deteriorate as result of protein inhibition caused by metronidazole and its metabolites binding to RNA.^16^

A limitation of this review is the lack of high‐level evidence and the small body of literature (which consists of only case reports and case series). It is therefore not possible to draw firm conclusions about the use of metronidazole in the treatment of VCD. In addition, metronidazole was used as an adjunctive treatment for CD alongside other systemic treatments. It is therefore difficult to draw firm conclusions on the effectiveness of metronidazole alone. However, we suggest that there are sufficient reported positive responses to warrant further investigation.

## 6 CONCLUSION

This systematic review suggests that metronidazole may be useful in managing VCD, either as a primary treatment or adjunctive therapy. However, the evidence is currently limited to case series and case reports which were insufficient to perform a meta‐analysis. Higher quality evidence in the form of randomised controlled trials is ideally needed to formally evaluate VCD treatment methodologies. Furthermore, better safety reporting in all published study types is needed. It is important to consider a diagnosis of VCD in patients with oedema, induration, ulcers and a variety of other presentations, in the anogenital area, even in the absence of GI CD. Management of VCD requires a multidisciplinary approach to optimise outcomes.

## CONFLICTS OF INTEREST

None to declare.

## AUTHOR CONTRIBUTIONS

**Nina Simon**: Conceptualization (Supporting), Data curation (Lead), Formal analysis (Lead), Investigation (Lead), Methodology (Lead). **Zahra Moledina**: Data curation (Equal), Formal analysis (Equal), Writing – original draft (Lead), Writing – review & editing (Lead). **Rosalind Simpson**: Conceptualization (Lead), Data curation (Supporting), Supervision (Equal), Writing – review & editing (Equal). **Lisa Kirby**: Conceptualization (Lead), Data curation (Supporting), Formal analysis (Equal), Supervision (Lead), Writing – review & editing (Equal).

## ETHICS STATEMENT

Not applicable.

## Supporting information

Supporting Information S1

## Data Availability

The data that support the findings of this study are available from the corresponding author upon reasonable request.
