# Supplementary material for: Metronidazole for the treatment of cutaneous vulval Crohn disease: A systematic review
Source: Skin Health Dis. 2023 Apr 26;3(3):e210. doi: 10.1002/ski2.210 (PMC10233082; doi:10.1002/ski2.210)
Supplement: Supplementary file 1 — Supporting Information S1 [file SKI2-3-e210-s001.docx]

# **Appendix 1. PRISMA 2020 Checklist**

| **Section and Topic** | **Item #** | **Checklist item** | **Location where item is reported** |
| --- | --- | --- | --- |
| **TITLE** | | |  |
| Title | 1 | Identify the report as a systematic review. | Pg 1, 2 |
| **ABSTRACT** | | |  |
| Abstract | 2 | See the PRISMA 2020 for Abstracts checklist. | Pg 2 |
| **INTRODUCTION** | | |  |
| Rationale | 3 | Describe the rationale for the review in the context of existing knowledge. | Pg 2, 3, 4 |
| Objectives | 4 | Provide an explicit statement of the objective(s) or question(s) the review addresses. | Pg 2, 3 |
| **METHODS** | | |  |
| Eligibility criteria | 5 | Specify the inclusion and exclusion criteria for the review and how studies were grouped for the syntheses. | Pg 5 |
| Information sources | 6 | Specify all databases, registers, websites, organisations, reference lists and other sources searched or consulted to identify studies. Specify the date when each source was last searched or consulted. | Pg 4, Appendix 2 |
| Search strategy | 7 | Present the full search strategies for all databases, registers and websites, including any filters and limits used. | Pg 4, Appendix 2 |
| Selection process | 8 | Specify the methods used to decide whether a study met the inclusion criteria of the review, including how many reviewers screened each record and each report retrieved, whether they worked independently, and if applicable, details of automation tools used in the process. | Pg 4, 5 |
| Data collection process | 9 | Specify the methods used to collect data from reports, including how many reviewers collected data from each report, whether they worked independently, any processes for obtaining or confirming data from study investigators, and if applicable, details of automation tools used in the process. | Pg 4, 5 |
| Data items | 10a | List and define all outcomes for which data were sought. Specify whether all results that were compatible with each outcome domain in each study were sought (e.g. for all measures, time points, analyses), and if not, the methods used to decide which results to collect. | Pg 5 |
|  | 10b | List and define all other variables for which data were sought (e.g. participant and intervention characteristics, funding sources). Describe any assumptions made about any missing or unclear information. | Pg 4, 5 |
| Study risk of bias assessment | 11 | Specify the methods used to assess risk of bias in the included studies, including details of the tool(s) used, how many reviewers assessed each study and whether they worked independently, and if applicable, details of automation tools used in the process. | 5 |
| Effect measures | 12 | Specify for each outcome the effect measure(s) (e.g. risk ratio, mean difference) used in the synthesis or presentation of results. | N/A |
| Synthesis methods | 13a | Describe the processes used to decide which studies were eligible for each synthesis (e.g. tabulating the study intervention characteristics and comparing against the planned groups for each synthesis (item #5)). | N/A |
|  | 13b | Describe any methods required to prepare the data for presentation or synthesis, such as handling of missing summary statistics, or data conversions. | N/A |
|  | 13c | Describe any methods used to tabulate or visually display results of individual studies and syntheses. | N/A |
|  | 13d | Describe any methods used to synthesize results and provide a rationale for the choice(s). If meta-analysis was performed, describe the model(s), method(s) to identify the presence and extent of statistical heterogeneity, and software package(s) used. | N/A |
|  | 13e | Describe any methods used to explore possible causes of heterogeneity among study results (e.g. subgroup analysis, meta-regression). | N/A |
|  | 13f | Describe any sensitivity analyses conducted to assess robustness of the synthesized results. | N/A |
| Reporting bias assessment | 14 | Describe any methods used to assess risk of bias due to missing results in a synthesis (arising from reporting biases). | Pg5 |
| Certainty assessment | 15 | Describe any methods used to assess certainty (or confidence) in the body of evidence for an outcome. | N/A |
| **RESULTS** | | |  |
| Study selection | 16a | Describe the results of the search and selection process, from the number of records identified in the search to the number of studies included in the review, ideally using a flow diagram. | Pg 5, 6, 7 |
|  | 16b | Cite studies that might appear to meet the inclusion criteria, but which were excluded, and explain why they were excluded. | Figure 1 (supplementary) |
| Study characteristics | 17 | Cite each included study and present its characteristics. | Appendix 3 |
| Risk of bias in studies | 18 | Present assessments of risk of bias for each included study. | Pg 7, Appendix 3 |
| Results of individual studies | 19 | For all outcomes, present, for each study: (a) summary statistics for each group (where appropriate) and (b) an effect estimate and its precision (e.g. confidence/credible interval), ideally using structured tables or plots. | N/A |
| Results of syntheses | 20a | For each synthesis, briefly summarise the characteristics and risk of bias among contributing studies. | N/A |
|  | 20b | Present results of all statistical syntheses conducted. If meta-analysis was done, present for each the summary estimate and its precision (e.g. confidence/credible interval) and measures of statistical heterogeneity. If comparing groups, describe the direction of the effect. | N/A |
|  | 20c | Present results of all investigations of possible causes of heterogeneity among study results. | N/A |
|  | 20d | Present results of all sensitivity analyses conducted to assess the robustness of the synthesized results. | N/A |
| Reporting biases | 21 | Present assessments of risk of bias due to missing results (arising from reporting biases) for each synthesis assessed. | Pg 7, Appendix 3 |
| Certainty of evidence | 22 | Present assessments of certainty (or confidence) in the body of evidence for each outcome assessed. | N/A |
| **DISCUSSION** | | |  |
| Discussion | 23a | Provide a general interpretation of the results in the context of other evidence. | Pg 7-9 |
|  | 23b | Discuss any limitations of the evidence included in the review. | Pg 9 |
|  | 23c | Discuss any limitations of the review processes used. | Pg 9 |
|  | 23d | Discuss implications of the results for practice, policy, and future research. | Pg 9 |
| **OTHER INFORMATION** | | |  |
| Registration and protocol | 24a | Provide registration information for the review, including register name and registration number, or state that the review was not registered. | Pg 4 |
|  | 24b | Indicate where the review protocol can be accessed, or state that a protocol was not prepared. | (Can be found on PROSPERO) |
|  | 24c | Describe and explain any amendments to information provided at registration or in the protocol. | (Can be found on PROSPERO) |
| Support | 25 | Describe sources of financial or non-financial support for the review, and the role of the funders or sponsors in the review. | N/A |
| Competing interests | 26 | Declare any competing interests of review authors. | Pg 1  (Can be found on PROSPERO) |
| Availability of data, code and other materials | 27 | Report which of the following are publicly available and where they can be found: template data collection forms; data extracted from included studies; data used for all analyses; analytic code; any other materials used in the review. | N/A |

# **Appendix 2. Search strategy to identify studies documenting metronidazole use in vulval CD**

| **MEDLINE ALL 1964 to 1^st^ August 2022** | **Embase 1974 to 1^st^ August 2022** |
| --- | --- |
| 1. exp vulva/ | 1. exp vulva/ |
| 2. vulv$.mp. | 2. vulv$.mp. |
| 3. exp genitalia/ | 3. exp genital system/ |
| 4. genit$.mp. | 4. genit$.mp. |
| 5. exp perineum/ | 5. exp perineum/ |
| 6. perianal.mp. | 6. perianal.mp. |
| 7. exp crohn disease/ | 7. exp crohn disease/ |
| 8. crohn$.mp. | 8. crohn$.mp. |
| 9. exp metronidazole/ | 9. exp metronidazole/ |
| 10. metronidazole.mp. | 10. metronidazole.mp. |
| 11. metro$.mp. | 11. metro$.mp. |
| 12. 1 OR 2 OR 3 OR 4 OR 5 OR 6 | 12. 1 OR 2 OR 3 OR 4 OR 5 OR 6 |
| 13. 7 OR 8 | 13. 7 OR 8 |
| 14. 9 OR 10 OR 11 | 14. 9 OR 10 OR 11 |
| **15. 12 AND 13 AND 14** | **15. 12 AND 13 AND 14** |

| **PubMed – Accessed 1^st^ August 2022** |
| --- |
| ("Vulva"[MeSH Terms] OR "vulv*"[Text Word] OR "Genitalia"[MeSH Terms] OR "genit*"[Text Word] OR "Perineum"[MeSH Terms] OR "perianal"[Text Word]) AND ("Crohn Disease"[MeSH Terms] OR "crohn*"[Text Word]) AND ("Metronidazole"[MeSH Terms] OR "metro*"[Text Word] OR "Metronidazole"[Text Word]) |

**Appendix 3**

| **Paper author, country, year of publication** | **Total patients**  **(n=)** | **Age of patients** | **Presence of Bowel CD?** | **Method of vuval CD diagnosis (Histology,**  **Clinical or**  **Both)** | **Duration of disease Vulval (and**  **GI if stated)** | **Vulval CD symptoms** | **Prior treatments** | **Concomitant treatments** | **MTZ Treatment regimen (including route)** | **Duration of MTZ treatment** | **Outcome - clinical/patient reported improvement** | **Duration**  **of follow up** | **Relapse rate** | **Adverse events** | **Other**  **comment** | **Quality assessment tool** | **Quality** |
| --- | --- | --- | --- | --- | --- | --- | --- | --- | --- | --- | --- | --- | --- | --- | --- | --- | --- |
| Ahad et al  UK  2017 (38) | 1 | 11 | Y | H | 1y | Induration  Erythema  Tender  Oedema | I&D - cyst  ABX  Top STR | IM | N/S | N/S | Significant improvement | N/S | N/S | N/S |  | JBI - CS | VL |
| Akutko et al  Poland  2017 (39) | 1 | 11 | Y | B | 3y | Oedema | ABX  Anti-P  Anti-His  Top STR | ASA  EEN  IM | N/S | N/S | Lesions in anogenital region disappeared | N/S | N/S | N/S | Biopsy showed no granulomas | JBI - CR | L |
| Al-Niaimi et al  Uk  2013 (40) | 1 | 13 | Y | B | 8y GI  9m V | Asymptomatic  Oedema  Peau d'orange, dusky coloured | N/S | IL STR | O | N/S | Clear improvement  after 3m | N/S | N/S | N/S |  | JBI - CR | VL |
| Andrade et al  Portugal  2015 (54) | 1 | 20 | N/S | B | 5y | Discomfort, pruritis and dyspareunia Erythema  Oedema  Pustules and  Ulcers | ABX  Anti-F | 40mg STR | 500mg TID | 1m | Lesions did not subside | N/S | N/S | N/S |  | JBI - CR | VL |
| Bhoyrul et al  Australia  1969 (81) | 1 | N/S | N/S | B | N/S | Oedema - 77%  Ulceration - 35%  Fissures - 39% | N/S | 0.1% Top IM | N/S | N/S | 1 patient failed to respond | N/S | N/S | N/S |  | JBI - CS | VL |
| Blasco-Alonso et  al  Spain  2016 (41) | 2 | 13  13 | Y | B | 2m to 3y | Oedema Abscess | N/S | ASA  EN  IM | N/S | NS | Complete disappearance of genital injury | N/S | N/S | N/S |  | JBI - CS | VL |
| Cantero et al  Spain  2021 (42) | 1 | 9 | Y | B | 12m | Non-tender Asymmetric oedema Erythema | N/S | 50 mg IM | 250mg daily | NS | Resolution | N/S | N/S | N/S |  | JBI - CR | L |
| Corbett et al  US  2010 (43) | 1 | 8 | Y | B | 9m | Bilateral oedema  Erythema | Top STR 5-ASA | N/S | O 10mg/kg/day | 2m | No further improvement | 6m | N/S | N/S |  | JBI - CR | VL |
| Das et al  India  2016 (20) | 1 | 30 | N | B | 2.5y GI  1y V | Oedema  Nodules | OTCs | 40 mg daily  STR | 400mg TID | 3m | No significant improvement | 3m | N/S | N/S | Inconclusive GI CD investigation | JBI - CR | VL |
| Duhra et al  UK  1988 (55) | 1 | 45 | N | B | 1m | Painful oedema | STR  Anti-inflam  Supp | N/S | O 800mg daily | 4m | Complete symptomatic and objective healing | +1y | No relapse for 1y | N/S |  | JBI - CR | L |
| Dural et al  Istanbul  2019 (44) | 1 | 5 | N | B | 7m | Oedema  Pruritus  Vaginal bleeding Foul smell discharge | N/S | 10mg/kg/dose  ABX twice daily | O 20mg/kg/d | 4m | Significant improvement after 10d and maintained after cessation | N/S | N/S | N/S | Some GI symptoms  but no  definitive  diagnosis of  GI CD | JBI - CR | L |
| Ferreira et al  Portugal  2016 (23) | 1 | 13 | Y | B | 6w | Painless asymmetric  Oedema  Erythema | Top STR  Anti-F | STR IM | N/S | N/S | Significant improvement 1y later | 1y | Relapse after 1y | N/S | CD diagnosed 1 year later | JBI - CR | VL |
| Fitzgerald et al  UK  2016 (56) | 1 | 52 | N | H | N/S | Severe pain Ulceration | Anti-TNF  STR | N/S | O Twice daily | N/S | No improvement | N/S | N/S | N/S |  | JBI - CR | VL |

| Holohan et al  Ireland  1988 (57) | 1 | 32 | N/S | B | 15m | Painful ulceration Offensive, irritating vaginal discharge | Top STR  STR ABX  Anti-F | N/S | 20mg/kg/day divided into 3 doses | 6m | Striking improvement within 3w | +2y | Relapse 2w after stopping treatment. Therapy resumed, then gradually tapered, asymptomatic for 6m | N/S |  | JBI - CR | L |
| --- | --- | --- | --- | --- | --- | --- | --- | --- | --- | --- | --- | --- | --- | --- | --- | --- | --- |
| Kazi et al  India  2013 (58) | 2 | 35  26 | N/S | B | N/S | Painful pruritus  Ulceration  Induration  Tender Oedema  Violaceous plaques | N/S | STR | O | N/S | Responded to treatment | N/S | N/S | N/S |  | JBI - CS | VL |
| Khaled et al  Tunisa  2010 (59) | 1 | 46 | N | B | 3y | Oedema  Erythema  Lesions  Ulceration | N/S | N/S | 1g/d | 6m | Almost complete healing | N/S | N/S | N/S |  | JBI - CR | VL |
| Kim et al  Korea  1992 (45) | 1 | 16 | Y | B | 3y GI  5m V | Erythema Oedema | N/S | STR 20mg daily  1% Top STR | O 1gm | N/S | Lesions had much  improved after 2m | N/S | N/S | N/S |  | JBI - CR | L |
| Kingsland et al  UK  1991 (60) | 1 | 59 | Y | B | 1y | Hypertrophy  Erythema  Oedema  Tender | Derm NN cream | N/S | 400mg BID | 3m | Symptomatic and objective healing | +1y | No relapse for 1y | N/S |  | JBI - CR | L |
| Kowsika et al  US  1991 (61) | 1 | 29 | Y | B | 10y | Erythema  Induration  Oedema | N/S | STR  IM  MTX | N/S | N/S | Failed to respond | N/S | N/S | N/S | MTZ outcome described within many other treatments | JBI - CR | VL |
| Kremer et al  Israel  1984 (62) | 1 | 18 | N | H | 4m | Pustular eruption  Oedema  Erosion  Rash | Anti-inflam | N/S | 250mg QDS | 4m | Complete disappearance of symptoms | +4m | N/S | N/S |  | JBI - CR | L |
| Kuloglu et al  Turkey  2008 (46) | 1 | 10 | Y | B | 8m GI  6m V | Skin tags  Asymptomatic  unilateral  hypertrophy Erythema  Oedema | ABX  Anti-F  Top Anti-F | N/S | N/S | 1m | Outcome not described | N/S | N/S | MTZ stopped after a month due to elevated liver enzyme levels | Biopsy showed no granulomas | JBI - CR | VL |
| Laftah et al  UK  2015 (10) | 5 | N/S | N/S | B | N/S | Pruritus - 41%  Oedema - 64%  Sore - 73%  19-bilaterla 3-unilateral | N/S | 2 pts - ABX | O | MTZ+ABX  = 5w  MTZ = 3m18m | 3 failures (MTZ only)  2 clinical effective  outcomes (MTZ and  ABX) | N/S | N/S | N/S |  | JBI - CS | L |
| Lestre et al  Portugal  2010 (63) | 1 | 21 | N/S | H | 1y | Polypoid skin tags  Erythema | Cryotherapy  STR  Anti-inflam  IM | N/S | N/S | N/S | Inadequate response | N/S | N/S | N/S | MTZ outcome described within many other treatments | JBI - CR | VL |
| Leu et al  US  2009 (64) | 1 | 43 | Y | B | 27y GI  1y V | Erythema erosions  Ulceration  Pruritus | IV STR  ABX  PG analogue  Anti-V | STR  ABX | IV then O | N/S | Symptoms completely resolved after 2m | N/S | 9m after discharge, relapse | N/S |  | JBI - CS | L |

| Levine et al  US  1982 (79) | 1 | 44 | Y | N/S | 10y | Secreting ulceration Sores  Oedema  Tender | ABX STR  IM  Zinc  Vit C | ABX 100mg  BID  STR 20mg  daily  IM  Zinc  Vit C | 250mg 5 times  daily | N/S | Entire perineum healed except for 1 small draining site | 6m | Exacerbation upon any tapering of dose | N/S |  | JBI - CR | L |
| --- | --- | --- | --- | --- | --- | --- | --- | --- | --- | --- | --- | --- | --- | --- | --- | --- | --- |
| Madnani et al  India  2011 (65) | 1 | 37 | N | B | 4y | Painful ulceration  Erythema  Dyspareunia | STR | N/S | 400 mg TID | 1y | Complete healing of ulcers | 1y 3m | N/S | MTZ caused peripheral neuropathy, discontinued, relapse upon stopping. Then treated with Anti-TNF, minimal improvement |  | JBI - CR | L |
| Martin et al  UK  1997 (66) | 1 | 24 | Y | C | 18m | Bilateral oedema No ulceration | STR | STR 30 mg daily | 400 mg TID | N/S | Completely asymptomatic | 2y | N/S | N/S |  | JBI - CR | L |
| Massa et al  Portugal  2015 (47) | 1 | 12 | N | B | N/S | Ulceration/lesions  Discharge  Fissures  Violaceous patch | N/S | STR IM  ABX | n/a | N/S | Initial good response, relapse after corticosteroid tapering | 10m | Relapse upon STR tapering | N/S |  | JBI - CR | VL |
| Mokhtari et al  Iran  2018 (67) | 1 | 61 | N/S | B | 9y | Ulceration  Erythema | N/S | Top STR | 250mg TID | N/S | Apparent healing and increased quality of life | N/S | Relapse upon tapering of MTZ and life stress.  Subsequent lesions not responsive to MTZ alone | N/S |  | JBI - CR | L |
| Morales et al  Argentina  2000 (48) | 1 | 13 | Y | B | 4m | Skin lesions &  tags  Erythema  Oedema | N/S | STR  0.5mg/kg/day  ASA  75mg/kg/day  Vitamins  Folic acid  Iron | 250 mg TID | N/S | Moderately effective | N/S | N/S | N/S |  | JBI - CR | VL |
| Moretta et al  Italy  2018 (68) | 1 | 22 | Y | B | 2w | Papules  Erythema  Oedema | N/S | Anti-inflam 2g  daily  Top STR  Fusidic acid  BID | 750mg daily | 2m | Complete clearance after 2m | +6m | No relapse | N/S |  | JBI - CR | L |
| Mun et al  Korea  2010 (24) | 1 | 10 | Y | H | 1m | Non tender  Unilateral oedema | ABX | N/S | N/S | 2m | Marked improvement | 1.5y | N/S | N/S | CD diagnosed after vulval presentation | JBI - CR | VL |
| Myagerimath et al  UK  2013 (69) | 1 | 42 | Y | C | N/S | Induration | ABX  Surgery | N/S | Long term | 6m | Skin lesions healed eventually | N/S | N/S | N/S |  | JBI - CR | VL |
| O Grady et al UK 2021 (80) | 1 | N/S | Y | N/S | 2y | Oedema | STR Anti-TNF | Anti-TNF | N/S | N/S | Suppressed vulval symptoms | N/S | N/S | N/S |  | JBI-CR | VL |
| Pampin et al Spain 2013 (70) | 1 | 32 | N | B | 7y | Painful ulceration | STR ABX IM Colchicine Anti-inflam | N/S | N/S | N/S | Unsatisfactory response | N/S | N/S | N/S | MTZ outcome described within many treatments | JBI-CR | VL |
| Pinna et al  Italy  2006 (49) | 1 | 10 | Y | B | 6m | Oedema  Violaceous  Erythema  Hardening  Dusky cyanotic discoloration | ABX | STR 20 mg daily  Iron  Folic acid  Top STR  ABX ointment | 250mg TID | N/S | Initial apparent improvement | N/S | Chronic relapsing course | N/S | CD diagnosed after vulval presentation | JBI - CR | L |
| Pousa Mertinez et al  Spain  2017 (21) | 1 | 35 | Y | B | 15y GI | Painful erythema and oedema | Two  intestinal surgeries Anti-TNF | Top STR BID | O 800mg daily | 2w | Clinical improvement | +6m | Occasional  flares | N/S |  | JBI - CR | L |
| Preston et al  UK  2006 (71) | 1 | 38 | N/S | B | 2y | Oedema and  fissures | Top STR | 30mg STR  IM  1.5mg/kg/daily | 600mg daily | 2w-3m | Almost completely healed over, reduced oedema after 2w | N/S | MTZ replaced with ciclosporin after 3m. Flare up once | N/S |  | JBI - CR | VL |
| Price et al  UK  1995 (72) | 1 | 19 | Y | C | N/S | Unilateral pruritus  Oedema  Occasional ulcers | ABX | N/S | N/S | N/S | Symptoms worsened | N/S | N/S | N/S | CD diagnosed after vulval presentation | JBI - CR | VL |
| Rodrigues et al  Portugal  2012 (73) | 1 | 20 | N | B | 2y | Discomfort  Pruritus  Dyspareunia  Erythema  Oedema | Top Anti-F | ABX 500mg  BID  40mg STR | 500mg TID | 8d | Significant improvement | N/S | Relapse upon STR weaning | N/S | Some symptoms of GI CD but no conclusive evidence | JBI - CR | L |
| Schlak et al  US  2016 (50) | 1 | 12 | Y | B | 2m GI | Discharge Ulceration | ABX  Anti-F  STR  Anti-TNF | Anti-TNF  STR  IM | N/S | N/S | Genital symptoms resolved after 5m | 5m | N/S | N/S |  | JBI - CR | VL |
| Schrodt et al  US  1999 (51) | 1 | 13 | Y | B | 6m | Erythema  Induration  Tender  Papules | STR  Anti-inflam  IM  Top ABX  Top STR | STR 30 mg daily Anti-inflam  250 mg TID  IM 75 mg daily  3% Top ABX  0.5% Top STR | O 250mg TID | Ongoing | Continues to experience exacerbations of her genital disease, necessitating hospitalization | N/S | N/S | N/S |  | JBI - CR | L |
| Schulman et al  US  1987 (74) | 1 | 23 | Y | B | 7y GI  2w V | Oedema  Induration  Tender  Pruritus  Erythema | ABX  Anti-F | STR 15mg IV every 6 hours | 250mg every 6 hours | 4 days | Normal appearance after 5m | +5m | N/S | N/S | MTZ begun as steroids were tapered. Initial dose given for 4 days then 1g/day | JBI - CR | L |
| Shields et al  US  2020 (52) | 3 | 15  31  31 | Y | B | 1y | Erythema  Pustules  Oedema  Pink scaly plaques  Pruritus | STR  Top IM  IM  Anti-TNF | ABX 500mg  every hour for 14 days  IM ointment  ABX ointment  Bleach baths  IL STR | 0.75% gel/cream pt3 had 1% cream  Top | N/S | Pt1 No improvement  Pt2 Resolved fissuring, pustules, and erythema Pt3 short term improvement | N/S | N/S | N/S |  | JBI - CS | L |
| Shukla et al  US  2020 (75) | 1 | 43 | Y | C | 10y GI  2y V | Pruritus Soreness | ABX  Anti-F | N/S | Repeated courses | N/S | Without benefit | N/S | N/S | N/S | MTZ described within many treatments | JBI - CS | L |
| Tuffnell et al  UK  1991 (53) | 1 | 10 | Y | B | 2-3m | Painful oedema  Induration  Fissures | N/S | N/S | N/S | N/S | Good clinical response | N/S | N/S | N/S | CD diagnosed after vulval presentation | JBI - CR | VL |
| Urbanek et al  UK  1996 (76) | 1 | 31 | Y | B | 2m | Oedema  Erythema  Induration | N/S | N/S | Top | N/S | Improved slowly | 2y | N/S | N/S |  | JBI - CS | VL |
| Vettraino et al  US  1995 (77) | 1 | 32 | Y | B | 3-4y | Warts  Deformities | Sitz bath  ABX | STR  Anti-inflam | O | N/S | Condition improved | N/S | N/S | N/S |  | JBI - CR | VL |
| Wylomanski et al  UK  2016 (78) | 1 | 29 | N/S | B | 7y | Oedema  Ulceration | N/S | STR 0.5 to 1mg/kg | 1g/day | N/S | Did not prevent the occurrence of numerous vulvar relapses | 4y | N/S | N/S |  | JBI - CR | VL |

**Key**

Y – yes

N – no

N/S – not stated

H – histology

C – clinical

B – both histology and clinical

y – years

m – months

w – weeks

d – days

MTZ - metronidazole

GI – gastrointestinal

V – vulval

CD – Crohn disease

I&D – incision and drainage

ABX – antibiotics

STR – steroids

Anti-P – anti-parasitic

Anti-his – anti-allergic

Anti-F – antifungal

Anti-inflam – anti-inflammatory

OTC – over the counter

ASA – aminosalylic acid

Supp – suppositories

(E)EN – (exclusive) enteral nutrition

PG – prostaglandin

IM – immunomodulator

Derm NN – dermovate neomycin/nystatin

Vit – vitamin

IL – intralesional

O - oral

Top – topical

IV - intravenously

BID – twice daily

TID – three times daily

JBI-CR – Joanna Briggs Institute checklist for case reports

JBI-CS – Joanna Briggs Institute checklist for case series

VL – very low

L - low
